# Variation in *Arabidopsis* flowering time associated with *cis*-regulatory variation in *CONSTANS*

**DOI:** 10.1038/ncomms4651

**Published:** 2014-04-16

**Authors:** Ulises Rosas, Yu Mei, Qiguang Xie, Joshua A. Banta, Royce W. Zhou, Gabriela Seufferheld, Silvia Gerard, Lucy Chou, Naeha Bhambhra, Jennifer Deane Parks, Jonathan M. Flowers, C. Robertson McClung, Yoshie Hanzawa, Michael D. Purugganan

**Affiliations:** 1 Department of Biology, Center for Genomics and Systems Biology, New York University, New York, New York 10003, USA; 2 Department of Crop Science, University of Illinois, Urbana-Champaign, Champaign, Illinois 61801, USA; 3 Department of Biological Sciences, Dartmouth College, Hanover, New Hampshire 03755, USA; 4 Department of Biology, The University of Texas at Tyler, Tyler, Texas 75799, USA

## Abstract

The onset of flowering, the change from vegetative to reproductive development, is a major life history transition in flowering plants. Recent work suggests that mutations in *cis*-regulatory mutations should play critical roles in the evolution of this (as well as other) important adaptive traits, but thus far there has been little evidence that directly links regulatory mutations to evolutionary change at the species level. While several genes have previously been shown to affect natural variation in flowering time in *Arabidopsis thaliana*, most either show protein-coding changes and/or are found at low frequency (<5%). Here we identify and characterize natural variation in the *cis-*regulatory sequence in the transcription factor *CONSTANS* that underlies flowering time diversity in *Arabidopsis*. Mutation in this regulatory motif evolved recently and has spread to high frequency in *Arabidopsis* natural accessions, suggesting a role for these *cis*-regulatory changes in adaptive variation of flowering time.

## Abstract (web summary)

The transcription factor *CONSTANS* regulates the timing of flowering in *Arabidopsis*. Rosas *et al.* report that genetic variation in the *cis*-regulatory regions of this gene contributes to natural phenotypic variation in flowering time.

## Graphical abstract

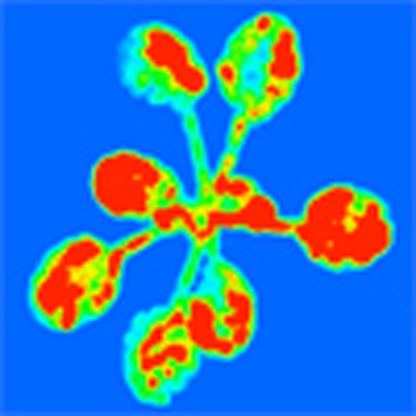

The onset of flowering, the change from vegetative to reproductive development, is a major life history transition in flowering plants. Flowering phenology is critically tied to the reproductive ecology of flowering plants, and is an important determinant of fitness in a variable environment^1^,^2^. Flowering time in *Arabidopsis thaliana*, a weedy annual plant, has a wide range of variation, and molecular genetic studies have elucidated many of the key pathways that plants utilize to sense seasonal cues, providing unique opportunities for investigators to examine the molecular genetic basis of flowering time variation in an ecological and evolutionary context.

*CO* is a photoperiod-dependent flowering time locus^3^ that encodes a zinc-finger transcription factor downstream of photoreceptor and circadian clock genes. Sequence analysis in 25 accessions identified a 7-bp insertion/deletion (indel) polymorphism in the *CO* promoter (Fig. 1a)^4^. This indel is in a variable number of tandem repeat region consisting of multiple repeats of the 7-bp sequence 5′-CTTTACA-3′ (Fig. 1b). Previous work has demonstrated that the *CYCLING DOF FACTOR 1* (*CDF1*), whose expression exhibits circadian oscillation with the highest peak during the day, represses daytime *CO* mRNA expression through direct binding to a *cis*-regulatory element (CTTT) in this 7-bp repeat in the *CO* promoter^5^.

Here we show that natural variation in the copy number of this *CO* indel tandem repeat is associated with variation in the developmental transition of flowering time in *Arabidopsis* accessions. We further show that variation in flowering time can be explained by differences in expression between the alleles. Our results suggest that increased copy number of this tandem repeat evolved recently and quickly spread in *Arabidopsis* populations, and may play a role in life history variation in natural environments.

## Results

### Variation and recent origin of a *CO* promoter polymorphism

**Figure 1:**
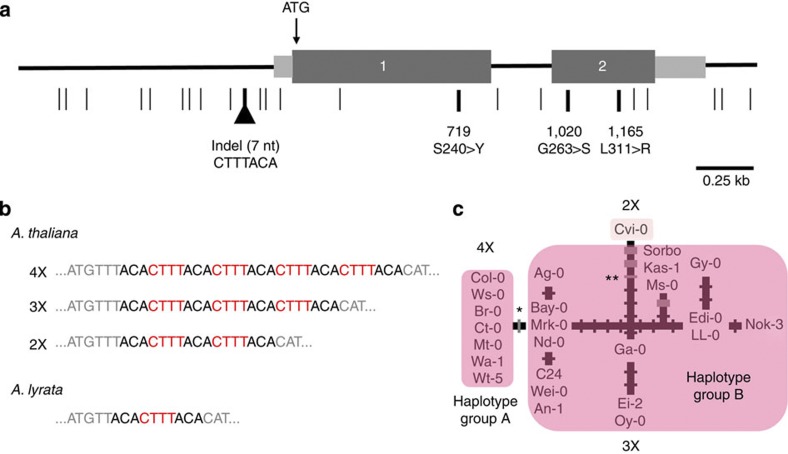
Molecular variation at the *CO* gene. (**a**) Schematic diagram of the *CO* gene showing relative locations of single nucleotide polymorphisms (SNPs) and indels. Numbered dark grey boxes indicate the exons, while light grey boxes indicate UTRs. The horizontal line indicates extent of sequenced region for *CO*. The translation start site is indicated by ATG. The light vertical lines are silent site SNPs and the thick vertical lines are nonsynonymous SNPs, which are shown with the amino-acid polymorphisms. The inverted triangle is the relative position of the 7-bp repeat motif at the *CO* promoter. (**b**) The sequence of the *cis*-regulatory CDF1-binding motif at the *CO* promoter (red cases), showing the variation between accessions that have various repeat numbers of the motif in *A. thaliana*. The sequence of the motif in an individual in *A. lyrata* is also shown. (**c**) Haplotype network of the *CO* gene. *A. thaliana* accessions that are found with 4X haplotype group A and 3X haplotype group B alleles are indicated. The thin tick marks are mutations, with the thick tick marks being nonsynonymous changes. The mutation that leads to an increase in the copy number of the 7-bp *cis*-regulatory repeat from 3X to 4X is indicated by an asterisk, while the decrease in copy number from 3X to 2X by two asterisks.

We observe allelic variation in a number of repeat units among the 25 *A. thaliana* accessions. Seven of the 25 accessions have four complete and one incomplete repeat of this 7-bp motif, resulting in four complete tandem copies of the DOF-binding site (4X). Seventeen accessions have three copies (3X) of the binding site, and one accession (Cvi-0) has two copies (2X) (Fig. 1c). We also sequenced the *CO* orthologue in *A. lyrata*, and we find that this sister species has only one complete copy as well as a truncated copy of this motif (TTACACTTTACA) (Fig. 1b). Using a haplotype network (Fig. 1c), we find that all seven alleles containing the 4X copies belong to a single monophyletic group with a single origin (haplotype group A or Hap A), while most of the other *A. thaliana* accessions have 3X copies (Hap B).

The only molecular difference between the haplotype group A and its ancestral haplotype in group B is the 7-bp insertion that results in an additional CDF1-binding site motif. We found that there are no other indels or single nucleotide polymorphisms that differentiate Hap A from Hap B among our 25 *A. thaliana* accessions (Fig. 1), suggesting a relatively recent origin of Hap A. Moreover, the alleles within the Hap A group contain no polymorphisms (nucleotide diversity *π*^6^=0) among the seven accessions, in contrast to the Hap B group that shows a moderately low level of nucleotide diversity (*π*=0.0025). Despite its apparent recent origin, the Hap A group is at relatively high frequency; ~41 percent of *A. thaliana* accessions have Hap A *CO* alleles, while 59 percent possess Hap B (Supplementary Table 1). Among the 271 accessions analysed, only Cvi-0 carries two repeats of the CDF1-binding site motif. Moreover, previous work using the pairwise haplotype sharing test has shown that the region around *CO* has an extended haplotype^4^, although compared with genome-wide data the magnitude of pairwise haplotype sharing around *CO* is only marginally significant at the 95th percentile level (Supplementary Fig. 1 in ref. 4). These features are consistent with a scenario of the recent evolution of the additional 7-bp indel at the *CO* promoter that has rapidly risen to high frequency across the species.

### *CO* promoter type is associated with flowering time variation

To examine whether the two most common *cis*-regulatory *CO* variants (4X and 3X promoter types) lead to natural variation in flowering time in natural accessions, we undertook a structured candidate gene association analysis. There is a significant association between *CO* promoter type and bolting time (restricted maximum likelihood (REML) mixed-model analysis of variance (ANOVA), *P*<0.0323) and rosette leaf number (REML mixed-model ANOVA*, P*<0.0375) under long days. Previous work indicates that the *FRIGIDA (FRI)* gene has a key role in repressing the vegetative–reproductive developmental transition, with both wild-type (delayed flowering) and loss-of-function alleles (early flowering) prevalent in natural populations^7^. We asked whether a genetic background that represses flowering (that is, *FRI*) has an effect on the promoter type variation. We found an epistatic interaction of the genetic background defined by the *FRI* alleles (wild-type or deletion), and *CO* promoter type for flowering time (REML mixed-model ANOVA, *P*<0.0027), but not in rosette leaf number (REML mixed-model ANOVA, *P*<0.1862). In this analysis, there is a significant difference in flowering time in the *FRI+* backgrounds (least square means estimate±s.e. is 52.44±4.82 for 4X versus 46.22±4.83 days for 3X), but no difference in *fri−* backgrounds (least square means±s.e. 43.35±4.72 for 4X versus 42.98±4.72 days for 3X). This association test suggests that delaying flowering (that is, in the presence of *FRI* functional alleles) is more likely to show the phenotypic outcomes of natural variation of the *CO* promoter type. However, these results should be interpreted cautiously, as the definition of *FRI* functional alleles is not predictive of activity^8^, and associations can arise from other epistatic interactions in different *A. thaliana* accessions^9^.

### *CO* promoter type affects flowering time in transgenic *Arabidopsis*

**Figure 2:**
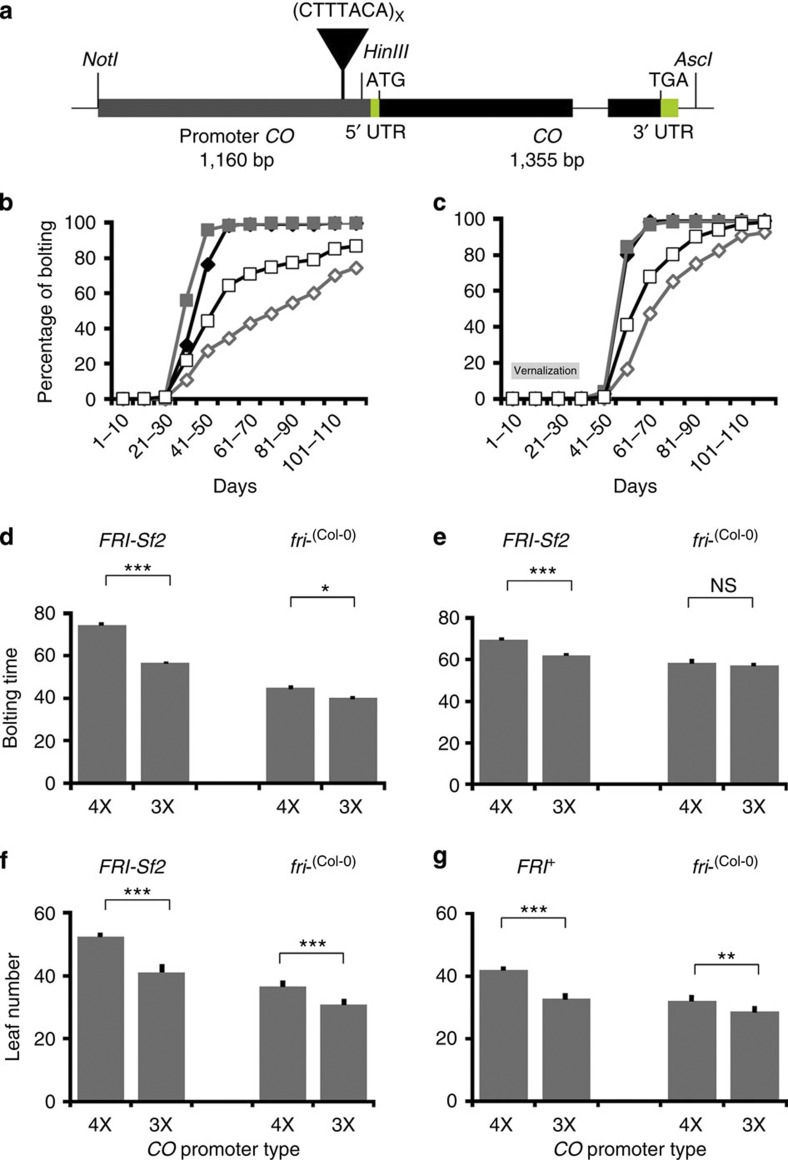
Phenotypes of *CO* transgenic complementation lines. (**a**) Diagram of the inserts for transgenic complementation lines that have a 3X or 4X *CO* promoter fused to *CO*-coding region: the promoter (grey block), the tandem repeat (inverted triangle), UTRs (green blocks), exons (black block) and an intron (thin line). (**b**–**c**) Cumulative flowering time of unvernalized (**b**) and vernalized (**c**) complementation lines of *CO* 3X (*n*=358 unvernalized plants, *n*=298 vernalized plants) and *CO* 4X (*n*=414 unvernalized plants, *n*=360 vernalized plants). Black diamonds indicate *CO* 4X *fri-*^(Col-0)^, grey squares *CO* 3X *fri-*^(Col-0)^, empty diamonds *CO* 4X *FRI-Sf2* and empty squares *CO* 3X *FRI-Sf2*. (**d**–**g**) Mean phenotypes in the *FRI-Sf2* and *fri−* backgrounds are shown in unvernalized (**d**,**f**) and vernalized (21 days at 4 °C) plants (**e**,**g**). The dark tick marks indicate the s.e. *Post hoc* Tukey’s test in REML mixed model ***P<0.0005, **P<0.005, *P<0.05. NS, not significant. Error bars indicate s.e.m.

To validate that the two most common *cis*-regulatory *CO* variants (4X and 3X promoter types) lead to functional differences in the ability to regulate flowering time, we created transgenic constructs of the Col-0 *CO* promoter fused to the Col-0 *CO* genomic region (Fig. 2a). We developed seven transgenic *Arabidopsis* lines of p*CO* (*4X)::CO FRI-Sf2 co-1*, three lines of p*CO* (*4X)::CO fri- co-1*, five lines of p*CO* (*3X)::CO FRI-Sf2 co-1* and three lines of p*CO (3X)::CO fri- co-1*. This transgene partially complements the *co-1*-mutant allele, which was expected, as the ~1 kb promoter region is unlikely to be the full promoter. Approximately 50 individuals from each of the 18 lines were grown in a fully randomized block design and scored for bolting time and rosette leaf number. The experiment was terminated after 122 days when all genotypes flowered, except for several plants carrying *FRI-Sf2* without vernalization (Fig. 2b,c). We analysed the data using a REML mixed model to test for *CO*, *FRI*, *CO* × *FRI* interaction, block and family effects (see Methods). In the analysis of bolting time, we find that the *FRI* genotype or *CO* transgenic promoter type effects are significant (REML mixed-model ANOVA, *P*<0.0001) for both vernalized and unvernalized plants. The analysis indicates that an increase in the number of the *cis*-regulatory repeat motif in the *CO* promoter from 3X to 4X leads to later flowering (Supplementary Table 2). For example, least squares mean estimate for bolting time is 48.46±0.93 days for the *CO* 3X promoter type and 59.65±0.93 days for the *CO* 4X promoter type in unvernalized genotypes (Supplementary Table 3). We also tested several *CO* 2X promoter constructs. Unlike the 3X and 4X transgenic lines, these showed greater variability in flowering times—early flowering in *fri*− backgrounds similar to that of 3X plants, but later flowering in *FRI-Sf2* lines similar to 4X constructs in unvernalized conditions (Supplementary Tables 4 and 5).

Our analysis also indicates an epistatic interaction between *CO* and the timing of the reproductive transition, in our case driven by variation in *FRI* in vernalized (REML mixed-model ANOVA, *P*<0.0001) and unvernalized (REML mixed-model ANOVA, *P*=0.0001) conditions. Thus, the effect of *CO* promoter type seems to be dependent on how fast the developmental transition occurs as determined by the *FRI* background (Fig. 2, Supplementary Table 2). In a *fri−* background, there is a marginally significant difference in bolting time between *CO* promoter types in unvernalized condition (*post hoc* Tukey’s test, *P*=0.02; Fig. 2d), and no significant differences in vernalized conditions (*post hoc* Tukey’s test, *P*=0.50; Fig. 2e). In contrast, the bolting time of *CO* 4X plants carrying *FRI-Sf2* was significantly later than *CO* 3X plants carrying *FRI-Sf2* both with and without vernalization (*post hoc* Tukey’s test, *P*<0.0001; Fig. 2d,e, Supplementary Table 3).

These patterns are consistent if we use rosette leaf number on bolting as a developmental surrogate for flowering time; the mean number of leaves for *CO* 4X plants are 44.58±0.52, while *CO* 3X has 36.00±0.53 leaves in unvernalized conditions (REML mixed-model ANOVA, *P*<0.0001). Moreover, unlike in bolting time, there is a significant difference in rosette leaf number between *CO* promoter types in *fri−* backgrounds in both unvernalized and vernalized conditions (*post hoc* Tukey’s test, *P*<0.0001; Fig. 2f,g; Supplementary Table 6). The transgenic results are consistent with the structured association results, where 4X *CO* promoter types showed later flowering in the *FRI*-active background.

### *CO* promoter type controls differences in gene expression

An obvious hypothesis is that variation in the number of the 7-bp indel motif may result in gene expression differences that lead to phenotypic variability in flowering time. We measured *CO* expression in 16 *Arabidopsis* accessions with combinations of *CO* and *FRI* allele types. A two-way ANOVA with interaction gave differences in *CO* expression, with twofold higher expression in the *CO* 4X allele (8.65±1.7) than accessions with *CO* 3X (4.22±1.1); these results, however, were marginally nonsignificant (ANOVA, *P*=0.0519). *FRI* allele type or the *CO–FRI* interaction gave no significant differences (Supplementary Tables 7 and 8).

*FRI* is an activator of the flowering time repressor *FLC*^10^,^11^, which is ~2 Mb distal from *CO* on chromosome V. Thus, we also measured *FLC* expression, and performed an analysis of variance (ANCOVA) of allele type with *FLC* expression with interaction term, and found that *FLC* expression does not explain the variation in *CO* expression (Supplementary Table 9). Moreover, linkage disequilibrium (LD) decays in about 10 kb in *Arabidopsis*^12^, and the LD between *CO* promoter type and *FLC* haplotype^13^ is low (*r*^2^=0.00021). This suggests that neither *FRI* nor *FLC* gene products are responsible for variation in *CO* expression, although the *CO* phenotypic effect might only be seen in a genetic background with delayed flowering (that is, *FRI*+).

**Figure 3:**
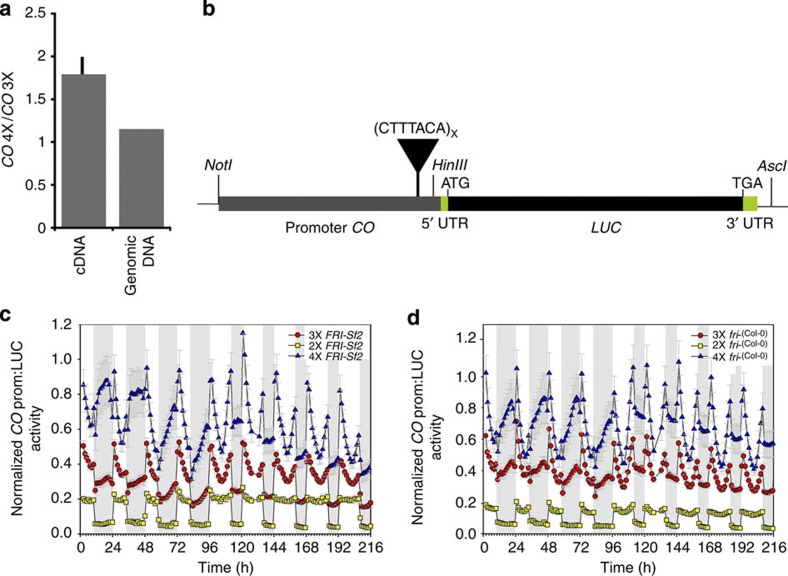
*CO* promoter type drives differences in gene activity. (**a**) Expression ratio in a semi-hybrid (Col-0) heterozygote genetic background measured with pyrosequecing. Genomic DNA was used to quantify PCR biases (*n*=7 plants). (**b**) Luciferase gene expression in transgenic reporter lines (see Fig. 2a legend). Luciferase expression was continuously monitored in seedlings in the first 96 h under short days, followed by 120 h in long days. Experimental periods of dark are shown by the light grey boxes. Expression levels of *LUC* for 2X (*n*=36 plants), 3X (*n*=60 plants) and 4X (*n*=60 plants) *CO* promoter reporter constructs are shown for (**c**) *FRI+* and (**d**) *fri−* backgrounds. Error bars indicate s.e.m.

To further test this, we measured relative levels of *CO* mRNA from *CO* 3X and 4X promoters in heterozygous plants^14^,^15^ in a *fri*− background. The *CO* 4X allele had ~1.5-fold higher expression levels than *CO* 3X allele (Fig. 3a). To study this expression difference in greater detail, we transformed *A. thaliana* Col-0 with p*CO* (*4X*, *3X* or *2X*)::*LUC* reporter gene constructs (Fig. 3b). *LUC* expression under control of *CO* 4X, 3X and 2X promoters was continuously monitored *in planta* for 120 h under long-day conditions after a 96-h entrainment under short days (Fig. 3c,d). *LUC* expression followed a circadian pattern under all three *CO* promoter types.

**Figure 4:**
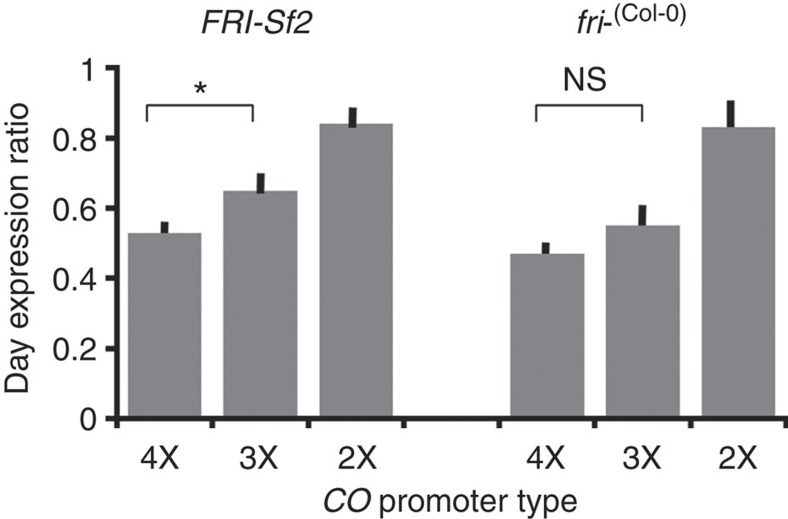
Variation in daytime repression of *CO*. Ratio of daytime minimum over dawn maximum expression of LUC reporter gene driven by different *CO* promoter types. Two-tailed *t*-test: *P<0.05. NS, not significant. Error bars indicate s.e.m.

The levels of *LUC* expression is always highest in p*CO(4X)::LUC* followed by p*CO(3X)::LUC* (Fig. 3c,d) in both *FRI-Sf2* and *fri−* backgrounds throughout the time course of the experiment, while expression of the p*CO(2X)*::*LUC* construct is consistently lower. Although overall *CO* expression increases with greater numbers of the binding site, there are nevertheless clear differences in the pattern of daytime *CO* expression from the different promoter types. We examined the ratio of the minimum *CO* expression level during the daytime to its maximum at dawn (Fig. 4). Therefore, based on our data, the simplest explanation is that the greater the number of the motif, the higher the daytime repression of the *CO.* The difference between *CO* 4X and 3X daytime repression is significant in the *FRI* background (*t*-test, *P*<0.026) but not in *fri−* (*t*-test, *P*<0.186). The expression pattern of the *CO* 2X is unusual, overall expression is low and there is very little repression of *CO* during the daytime (Figs 3 and 4).

## Discussion

We identified *cis-*regulatory variation in *CO*, a gene that plays a key role in the flowering time developmental transition. The sequence variation in the promoter region seems to have evolved relatively recently, and is responsible for differences in flowering time and gene expression under laboratory conditions. This *CO* promoter element may orchestrate a complex series of regulatory interactions. While this motif at the *CO* promoter binds the Dof transcription factor CDF1, there are also multiple examples in which Dof factors interact with other types of transcription factors, such as basic leucine zipper proteins and MYB proteins, and modulate their binding with DNA^16^. It is also possible that the *CO* 4X/3X promoter variation affects other nearby binding sites of transcriptional activators of *CO*, such as the case of FHB transcription factors^17^. Moreover, the protein GIGANTEA^18^,^19^ binds to this region of the *CO* promoter to facilitate CDF1 degradation and thus increase *CO* expression^20^,^21^. Together, these suggest a complex interplay between activators and repressors on this *cis*-regulatory element, which might give subtle phenotypic outcomes in an early-flowering background, but larger phenotypic outcomes in genetic backgrounds with delayed reproductive transition (that is, *FRI* and *FLC*-active backgrounds). The molecular mechanisms that connect these regulatory interactions to flowering time phenotypes require further study, but it is clear that natural variation in this *cis*-regulatory motif has a significant effect on both *CO* expression and flowering time phenotype.

Although it has been argued that *cis*-regulatory mutations underlie much of phenotypic evolution^14^,^22^,^23^, there are as yet relatively few functionally validated examples of identified *cis-*regulatory changes that are known to have high frequency in natural populations. In the case of *FRI*, the natural allelic variation results functional variation in the protein-coding region or regulatory sequences^7^. Our work now provides evidence for the role of a high-frequency *CO* promoter mutation in flowering time variation in *A. thaliana*, which was not observed on the observed coding region mutations. It remains to be seen whether the role of the *CO cis-*regulatory variation we observe in controlled laboratory conditions extends to natural environments in the wild. Comprehensive analyses of gene expression^24^ and phenotype in the field^25^, as well as geographical allele distributions across the species range^26^, will be crucial in further understanding the evolutionary and ecological significance of this promoter polymorphism. Nevertheless, our study further supports the importance of modulation of gene expression levels in quantitative phenotypic variation and possibly in evolutionary diversification, and demonstrates that gene regulatory mutations can play important roles in life history diversification in this model wild plant species.

## Methods

### Plant material and growth conditions

*A. thaliana* natural accessions used in this study were obtained from the Arabidopsis Biological Resource Center (Ohio, USA). *A. lyrata* seed was provided by O. Savolainen (Oulu, Finland). The *co-1*-mutant line that is introgressed in Col-0 was provided by G. Coupland (Koln, Germany). The *FRI Sf2* line carrying the *FRI* locus that is introgressed from Sf2 into Col-0, and the *FRI Sf2 co-1* line carrying the *FRI* locus from Sf2 and the *co-1* mutation were provided by R. Amasino (Madison, USA).

Plants were grown under continuous fluorescent light, long-day (14L:10D) or short-day (10L:14D) conditions at 21 °C on Metromix 360 soil (SUNGRO Horticulture, USA). For screening transgenic plants, plants were grown under continuous fluorescent light on 1.0% (w/v) agar plates containing MS salts (Sigma), 2% sucrose and 50 μg ml^−1^ kanamycin after sterilization of the surface of the seeds.

### DNA sequence analysis

Twenty-four accessions were used for sequencing the *CO* locus, including ~1 kb promoter region, 61 bp 5′ UTR, the coding region, 160 bp 3′ UTR and ~300 bp 3′ flanking region (Genbank accession numbers GQ176989-GQ177012)^4^. All sequences were assembled using Phred/Phrap (CodonCode, Dedham, MA) and aligned against the Col-0 *CO* sequence using BioLign2.0.9 (Tom Hall, Ibis Therapeutics, Carlsbad, CA). Levels of nucleotide diversity per silent site (*π*)^6^ and the Watterson’s population mutation parameter *θ*_w_^27^ were estimated using DNASP4.1^28^. Haplotype tree was constructed using a maximum parsimony analysis in PAUP*, with a heuristic search and stepwise addition^29^. One insertion/deletion in the promoter region was treated as a single change.

### Genotyping analysis

Genotyping for the repeat number variation of the Dof *cis*-element in the *CO* promoter was carried out using the PCR primers COSSLP01F (5′-GCAAGTGGCAAAACCTAAGC-3′) and COSSLP01R (5′-GAGAGAATAAGTAGAGGGGAGATCG-3′) with the PCR condition of 1 cycle at 94 °C for 4 min, 32 cycles for 94 °C 30 s, 55 °C 30 s and 72 °C 30 s, and a final extension step at 72 °C for 5 min. The PCR products were visualized on 3% agarose gel with EtBr staining. The nonsynonymous substitution L311>R identified in the *CO-*coding region at position 1,165 bp from ATG was genotyped by PCR using the PCR primers CO1165F (5′-CAAGCCAGATGATAACAGTAACAC-3′) and CO1165R (5′-TTCTCAAATTTCCTTGTCTTCCTC-3′) with the PCR condition described above, followed by *Bsp*EI restriction enzyme digestion and visualization on 2% agarose gel.

### Candidate gene association and LD tests

Structured Association Mapping^30^ was performed using JMP Genomics version 5.0 (SAS Institute, Cary, NC) using 264 accessions that were phenotyped for flowering time with no vernalization treatment under long day and short day^31^,^32^. A mixed model was used for testing for genotype–phenotype association to control for population structure and kinship within the sample. These analyses, including population stratification and kinship analyses, are carried out as described previously^32^. To measure LD between *CO* promoter types and *FLC* types (*r*^2^), we used the Measure.R2 function of the LDcorSV package^33^ in R^34^.

### Plasmid construction and plant transformation

For LUC expression analysis under the natural repeat variation of the Dof *cis*-element in the *CO* promoter, a genomic fragment of 1 kb upstream from the *CO* transcription start site was amplified from Col-0 genome using the PCR primers COP02F (5′-CCGCGGCCGCTTAAATTGCATCTATGGATCATGTGGACTA-3′) and COP02R (5′-CCGGCGCGCCTAATAACTCAGATGTAGTAAGTTTGATGGT-3′) containing NotI and AscI restriction sites, respectively, in the 5′ end. The following PCR conditions were used: 94 °C for 4 min, 28 cycles for 94 °C for 30 s, 55 °C for 30 s, 72 °C for 2 min and a final step at 72 °C for 8 min. The PCR fragments were cloned into pCR2.1 vector (Invitrogen), resulting in the *CO* promoter p*CO* (4X) that carries 4X repeat Dof *cis-*element. For engineering *CO* promoters that carry 3X and 2X Dof *cis*-element, the first set of PCR amplifications was performed using the COP02F primer and the reverse primers COP4mR (5′-GCCTATGTGTAAAGTGTAAAGTGTAAAGTGTAAACATCTC-3′) or COP3mR (5′-GGAAGGCCTATGTGTAAAGTGTAAAGTGTAAACATCTCCT-3′), which contain artificial mutations in the repeat number of the Dof *cis*-element with 3X and 2X, respectively. The second set of PCR amplifications was performed using the forward primers COP4mF (5′-GAGATGTTTACACTTTACACTTTACACTTTACACATAGGC-3′) or COP3mF (5′-AGGAGATGTTTACACTTTACACTTTACACATAGGCCTTCC-3′), which contain artificial mutations in the repeat number of the Dof *cis*-element with 3X and 2X, respectively, and the reverse primer COP02R. The first and second PCR products carrying the same repeat number of Dof *cis*-element (3X or 2X) were combined and used as templates for the third round of PCR amplification with COP02F and COP02R primers, and cloned into pCR2.1, resulting in p*CO (3X)* and p*CO (2X)*. After verification of the inserted fragments by sequencing, p*CO (4X)*/pCR2, p*CO (3X)*/pCR2 and p*CO (2X)*/pCR2 were digested by NotI and AscI, and the digested fragments were subcloned into NotI-AscI-digested pENTER/D-TOPO vector (Invitrogen), resulting in p*CO (4X)*/pENTER, p*CO (3X)*/pENTER and p*CO (2X)*/pENTER. The resulting clones were further subcloned into a binary vector pKGWL7 (University of Ghent, Ghent, Belgium) using the Gateway cloning system following the manufacturer’s protocol (Invitrogen), resulting in p*CO (4X)::LUC*, p*CO (3X)::LUC* and p*CO (2X)::LUC*.

To generate complementation constructs, the full-length genomic region of *CO* including ~180 bp upstream region from the ATG and 3′ UTR was first amplified using Col-0 genomic DNA (gDNA) as a template with COF primer (5′-CAAAAGCTCAACTAGCTGCAAGAGGATCCAATA-3′) and COR primer (5′-AAGGCGCGCCATATTTCACAAATGGGGATCTGTACC-3′) that contains an AscI restriction site, and cloned into pCR2.1. After checking the sequence of the inserted fragment, the HinIII site located ~70 bp upstream from the ATG of the *CO* promoter and the AscI site located in the COR primer were used to digest and subclone the *CO* genomic fragment into HinIII-AscI-digested p*CO (4X)*/pENTER, p*CO (3X)*/pENTER and p*CO (2X)*/pENTER to combine the *CO* genomic region with the *CO* promoter constructs. The resulting clones were further subcloned into the binary vector pKGWL7 using the Gateway cloning system, resulting in p*CO (4X)::CO*, p*CO (3X)::CO* and p*CO (2X)::CO*. These clones were used to transform the *Agrobacterium* strain GV3101 by electroporation.

Transformation of *Arabidopsis* was performed with the floral dip method using the *Agrobacterium* strain GV3101 transformed with p*CO (4X)::LUC*, p*CO (3X)::LUC*, p*CO (2X)::LUC*, p*CO (4X)::CO*, p*CO (3X)::CO* and p*CO (2X)::CO*. The obtained *Agrobacterium* lines carrying p*CO (4X)::LUC*, p*CO (3X)::LUC* or p*CO (2X)::LUC* were used to transform Col-0 plants, and the lines carrying p*CO (4X)::CO*, p*CO (3X)::CO* or p*CO (2X)::CO* were used to transform *co-1*-mutant plants. Three independent transformations were carried out for each construct. Transformed plants were screened in MS agar plates containing kanamycin in the T1 generation. Approximately 30 kanamycin-resistant T1 plants per construct were identified. These transgenic plants were transferred onto soil and T2 seeds were harvested by selfing. Transgenic lines that segregated in 3:1 ratio for kanamycin resistance were identified in the T2 generation as single-insertion lines. To facilitate clarification of the effect of FRI on *CO* promoter activity, 10 T1 plants per construct carrying single insertion of *pCO (4X)::LUC*, *pCO (3X)::LUC* or *pCO (2X)::LUC* were crossed with *FRI-Sf2* plants in Col-0, and F1 seeds were selected for kanamycin resistance on MS agar plates. *FRI* genotypes were tested by PCR for the absence of a characteristic 16 bp deletion in Col-0 *fri-* in the F2 generation using the primers UJ26 (5′-AGATTTGCTGGATTTGATAAGG-3′) and UJ34 (5′-ATATTTGATGTGCTCTCC-3′), and homozygous lines for transgenes were identified in the F3 generation, resulting in five lines each for p*CO* (*4X*)*::LUC FRI-Sf2* and p*CO* (*4X*)*::LUC fri-*, five lines each for p*CO* (*3X*)*::LUC FRI-Sf2* and p*CO* (*3X*)*::LUC fri-*, and three lines each for p*CO* (*2X*)*::LUC FRI-Sf2* and p*CO* (*2X*)*::LUC fri-*. Similarly, 7 T1 plants carrying single-insertion p*CO (4X)::CO* and 10 T1 plants carrying single-insertion p*CO (3X)::CO* were crossed with *FRI-Sf2 co-1*, and kanamycin-resistant F1 plants were selfed for F2 seeds. F2 plants were then screened for plants carrying the genotype p*CO (3X* or *4X)::CO*, *co-1/co-1*, *FRI-Sf2/fri-.* F3 plants obtained from these plants were screened further for homozygous lines for transgenes, resulting in seven lines p*CO* (*4X)::CO FRI-Sf2 co-1*, three lines p*CO* (*4X)::CO fri- co-1*, five lines p*CO* (*3X)::CO FRI-Sf2 co-1* and three lines p*CO (3X)::CO fri- co-1*.

### Expression analyses in *Arabidopsis* accessions

A set of 16 accessions that have been previously studied for natural variation in vernalization^8^ were chosen to measure *CO*, *FLC* (At5g10140) and *β-actin* (At3g18780) expression. Seeds were sown on plates (14–16 seeds per plate) on MS Basal Salt Mixture (Sigma), MES Sodium salts 0.05% (Gibco BRL), sucrose 0.2% and agar 0.8% (Bacto Agar BD). Every *Arabidopsis* accession was grown in 3–4 plates, and the pool of plants within each plate was considered a biological replicate. Plates were kept at 4 °C in the dark for 4 days and then transferred to a growth chamber (Percival Scientific). To mimic the growing conditions of the LUC assay, we grew the plants on a short-day condition (10L/14D) for 7 days and then switched them to a long-day condition (16L/8D) for 4 days. Pools of plant tissue were harvested at the start of the light period, when *CO* expression is the highest (Fig. 3c,d). Total RNA was extracted with the Plant RNeasy kit (Qiagen), DNA was depleted with DNAse I Amplification Grade (Invitrogen) and single-stranded cDNA synthesized using Superscript III (Invitrogen). We used the COfor (5′-GAGAAATCGAAGCCCGAGGAGCA-3′) and COrev (5′-TCAGAATGAAGGAACAATCCCATA-3′) for *CO*, and Actinfor (5′-TGTCGCCATCCAAGCTGTTCTCT-3′) and Actinrev (5′-GTGAGACACCATCACCAGAAT-3′) for *β-actin*^35^. For *FLC*, we used the primers 5′-ATGGGAAGAAAAAAACTAGAAATCAA-3′ and 5′-CTAATTAAGTAGTGGGAGAGTCAC-3′^36^. Quantitative PCRs were performed in triplicate for each sample, using the LightCycler FastStart DNA Master SYBR Green I Version 17 (Roche), in a LightCycler 480 (Roche), and annealing temperature of 60 °C. *CO* and *FLC* expressions were quantitated using the 2^−Δ*C*t^ method using *β-actin* as reference gene. *CO* expression variation as a response of *CO* and *FRI* allele types was tested using a two-way ANOVA *CO*_expression_=*CO*_type_+*FRI*_allele_+CO*FRI+*ε*. FLC effect on *CO* expression was tested using the ANCOVA *CO*_expression_=*CO*_type_+*FLC*_expression_+CO*FLC+*ε*.

### Expression analysis in a Col-0 heterozygous background

A near isogenic line of Col-0 introgressed with *CO* 3X from the accession Ler was generated. A backcross heterozygote for 3X/4X was self-crossed to obtain the segregating genotypes 3X/3X, 3X/4X and 4X/4X. Seeds were disinfected in ethanol-bleach-water 4:1:3 and rinses of sterile water, and plated on MS media (Sigma-Aldrich) supplemented with sucrose 0.1% (Gibco BRL) and agar 1% (Bacto Agar BD), and plates were kept at 4 °C for 4 days in the dark. Thereafter, seedlings were grown at 22 °C, long day (16L:8D) (Percival Scientific) for 15 days, and the shoots harvested at 15 h of light period. Total RNA and gDNA were co-extracted (Promega SV Total RNA Isolation System). RNA was depleted from the RNA sample with RNAse-free DNAse (Promega) and single-stranded cDNA obtained using Superscript III (Invitrogen). The 265-bp PCR product spanned a polymorphism C/T at +297 bp from the *CO* start codon in Col-0 sequence. This was obtained using the primers 5′-TCAAGTTCACTCTGCCAATCG-3′ and (5′biotinylated)-TCTCTTCTCTGGATCGGTCATT-3′ following the PCR programme at 94 °C 3 min, (94 °C 30 s, 55 °C 30 s, 72 °C 30 s) × 40, 72 °C 3 min. The relative amount of C/T quantified using the sequencing primer 5′-CAAACCCACTTGCTAGA-3′ was quantified in both the gDNA to calculate PCR biases and the cDNA to estimate differences in *CO* allele expression. The PCRs were done in quadruplicate for each of the seven seedlings identified to be *CO* 3X/4X heterozygotes, and prepared for pyrosequencing using the PSQ-kit (Biotage) and assayed in a PSQ-96 sequencer (Biotage).

### LUC expression analysis

Single-insertion-independent homozygous transgenic plants that carry p*CO (4X)::LUC* (five lines), p*CO (3X)::LUC* (five lines) or p*CO (2X)::LUC* (three lines) in combination with *FRI-Sf2* or *fri-* were obtained as described above. Twelve seedlings per line were sterilized and grown on MS agar plates^37^ for 16 days after germination under short day (10L:14D), and transferred into individual wells of 96-well microtiter plates containing substrates for LUC. The plants were subjected to long day (16L:8D) in the TopCount luminometer (Perkin Elmer Life Sciences) for the following 7 days and expression levels of LUC were monitored continuously.

### *CO* allele complementation tests

Single-insertion homozygous transgenic *co-1* plants that carry p*CO (4X)::CO*, p*CO (3X)::CO* or p*CO (2X)::CO* in combination with *FRI-Sf2* or *fri-* were obtained as described above. Nine to 15 independent transgenic lines for each construct were crossed with *FRI Sf2 co-1*, and F1 seeds were selected for kanamycin resistance on MS agar plates. Plants carrying single-insertion transgene were selected by kanamycin resistance and *FRI* genotypes tested by simple sequence length polymorphism (SSLP) PCR for the absence of a characteristic 16-bp deletion in Col-0 *fri-* in the F2 generation. *co-1*-mutant allele was genotyped by PCR using the primers 5′-GCTCCCACACCATCAAACTT-3′ and 5′-TGGTACGCTGCAGTTTTGTT-3′, followed by Bfa-1 restriction to detect the presence or absence of the 9-bp deletion. Homozygous lines for transgenes were identified in the F3 generation. The presence of the transgene was confirmed by nested PCR using the primers on the pKGWL7 vector (5′-ATAGCTTCTGCCAACCGAAC-3′ and 5′- AACGCGCAATAATGGTTTCT-3′) for the first PCR, and the COSSLP01F and COSSLP01R primers for the second PCR, followed by Sanger sequencing of the purified second PCR product.

Five lines of p*CO(3X)::CO FRI+*, seven lines of p*CO(4X)::CO FRI+*, three lines of p*CO(3X)::CO fri−* and three lines of p*CO(4X)::CO fri-* were used for phenotyping. Seeds from homozygous lines were soaked in water for 4 days at 4 °C in the dark and sown in soil (Metro-Mix 360, SUNGRO Horticulture, USA). Seven days after sowing and growth in 22 °C long day (16L:8D; 2–4 leaf stage), 53–54 seedlings of each genotype were transplanted to 72-plug trays in a fully randomized design in 20 trays (blocks) for each experiment (vernalized and unvernalized). The unvernalized set was continuously grown in long-day condition (16L:8D) at 22 °C, 70% humidity in a walk-in chamber at the Center for Genomics and Systems Biology (New York University). The vernalized set was grown for 21 days at 4 °C in short-day conditions (8L:16D) and then transferred to the long-day chamber. Once a week we randomized the position of the trays within the chamber, and watered the plants with 1.5 l per tray. Bolting date and rosette leaf number were recorded at bolting. Number of primary leaves was also scored every week to avoid underestimating rosette leaf number due to senescence of old leaves. The experiment was terminated 124 days after sowing when the mean bolting per family was 75% in the unvernalized set, and 95% in the vernalized set. Two lines of *CO 4X FRI-Sf2* and one line of p*CO (3X) fri-* in the unvernalized set were not considered for further analyses, as the bolting was <10%. Correspondingly, only one line of p*CO (4X) FRI-Sf2* was not used in the analysis because bolting was <65% in the vernalized set. The data were analysed separately for the vernalized and the unvernalized sets, using a REML mixed model having the *CO* (two levels) and *FRI* (two levels) genotypes as fixed effects, genotype families nested within *CO* and *FRI* as fixed effects and the block as a random effect, according to the following model: *y*_phenotype_=*CO*_type_+*FRI*_allele_+CO*FRI+family[*CO*,*FRI*]+block_(random)_+*ε*. To include the 2X allele (Supplementary Tables S4 and S5), separate analyses were done adding a level to the *CO* term in the model. Statistical analyses were carried out in JMPGenomics 5.1 (SAS Institute Inc., USA). *Post hoc* Tukey’s test analysis was used to identify significant differences between groups.

## Author contributions

Y.H. and M.D.P. conceived the research; Y.H. and J.M.F. performed the sequence diversity analyses; J.A.B. and J.D.P. performed the association analysis; Y.H., U.R., Y.M., R.W.Z., J.A.B., S.G., L.C., N.B. and G.S. generated the transgenic lines; Q.X. and C.R.M. performed the LUC activity assays; U.R. and R.W.Z. performed the qPCRs and pyrosequencing assays; U.R. and R.W.Z. obtained the phenotypic complementation data; Y.H. and M.D.P. directed the research; and U.R., Y.H. and M.D.P. wrote the manuscript.

## Additional information

**Accession codes:** *CO* sequences of 24 *Arabidopsis* accessions: GQ176989-GQ177012.

**How to cite this article:** Rosas, U. *et al.* Variation in *Arabidopsis* flowering time associated with *cis*-regulatory variation in *CONSTANS*. *Nat. Commun.* 5:3651 doi: 10.1038/ncomms4651 (2014).

## Supplementary Material

Supplementary Information Supplementary Tables 1-9
