# Supplementary material for: Variation in Arabidopsis flowering time associated with cis-regulatory variation in CONSTANS
Source: Nat Commun. 2014 Apr 16;5:3651. doi: 10.1038/ncomms4651 (PMC3997802; doi:10.1038/ncomms4651)
Supplement: Supplementary Information — Supplementary Tables 1-9 [file ncomms4651-s1.pdf]

Supplementary Table 1. ***CO*** **genotypes of 271 accessions genotyped for the 7 bp indel.**

Accession name	Accession number	promoter type	L311>R substitution	Accession name	Accession number	promoter type	L311>R substitution
Aa-0	CS6600	3X	L	Bu-11	CS6641	4X	
Abd-0	CS932	3X	L	Bu-14	CS6646	3X	L
Ag-0	CS901	3X	L	Bu-19	CS6650	3X	L
Ak-1	CS6602	3X	L	Bu-2	CS6633	3X	L
An-1	CS6603	3X	L	Bu-20	CS6651	4X	
An-2	CS6604	3X	L	Bu-24	CS6655	3X	L
Ang-0	CS6605	3X	L	Bu-25	CS6656	3X	L
Ang-1	CS6606	3X	L	Bu-3	CS6634	3X	L
Bay-0	CS6608	3X	L	Bu-5	CS6636	3X	L
Bch-1	CS6609	3X	L	Bu-6	CS6637	3X	R
Bch-3	CS6610	3X		Bu-8	CS6639	4X	
Bch-4	CS6611	4X		Bu-9	CS6640	3X	L
Bd-0	CS6612	4X		Ca-0	CS6658	4X	
Be-1	CS6614	3X	L	Cal-0	CS6659	3X	L
Ber	CS3109	4X		Chi-0	CS6664	4X	
Bl-1	CS6615	3X	L	Chi-1	CS6665	4X	
Bla-1	CS6616	4X		Co	CS3180	3X	
Bla-10	CS6622	4X		Co-1	CS6669	3X	L
Bla-11	CS6623	4X		Co-3	CS6671	3X	L
Bla-12	CS6624	4X		Co-4	CS6672	3X	L
Bla-3	CS6618	4X		Col-0	CS6673	4X	
Bla-4	CS6619	3X	L	Ct-1	CS6674	4X	
Bla-6	CS6621	4X		Da-0	CS6676	4X	
Br-0	CS6626	4X		Db-0	CS6677	4X	
Bs-1	CS6627	3X	L	Db-1	CS6678	4X	
Bs-2	CS6628	3X	L	Di-0	CS6680	4X	
Bs-5	CS6629	3X	L	Di-1	CS6681	3X	L
Bsch-0	CS6630	4X		Di-G	CS910	3X	L
Bsch-2	CS6631	4X		Di-M	CS919	4X	
Bu-0	CS6632	4X		Do-0	CS6683	3X	L

Supplementary Table 1. ***CO*** **genotypes of 271 accessions genotyped for the 7bp indel (continuation).**

Accession name	Accession number	promoter type	L311>R substitution	Accession name	Accession number	promoter type	L311>R substitution
Dr-0	CS6684	3X	L	Gu-0	CS6730	3X	L
Dra-0	CS6685	4X		Gu-1	CS6731	4X	
Dra-1	CS6686	4X		Gy-0	CS6732	3X	L
Edi-0	CS6688	3X	L	H55	CS923	4X	
Ei-2	CS6689	3X	L	Ha-0	CS6733	4X	
Ei-4	CS6690	4X		Hi-0	CS6736	4X	
Ei-6	CS6692	4X		Hl-3	CS6904	4X	
Eil-0	CS6693	3X	L	Hl-2	CS6738	3X	L
El-0	CS6694	3X	L	Hn-0	CS6739	3X	L
Ema-1	CS6923	3X	L	Hs-0	CS6905	3X	L
En-1	CS6695	4X		Is-0	CS6741	4X	
En-D	CS920	4X		Is-1	CS6906	3X	L
Er-0	CS6698	4X		Je-0	CS6742	3X	L
Est-0	CS6700	4X		Je54	CS924	4X	
Fe-1	CS6703	3X	L	Jl-2	CS6744	3X	L
Fi-0	CS6704	4X		Jl-3	CS6745	4X	
Fi-1	CS6705	3X	L	Jl-5	CS6747	4X	
Fr-2	CS6708	3X	L	Jm-0	CS6748	3X	L
Fr-3	CS6709	4X		Jm-1	CS6749	4X	
Fr-4	CS6710	4X		Jm-2	CS6750	4X	
Fr-6	CS6712	4X		Ka-0	CS6752	3X	L
Fr-7	CS6713	4X		Kb-0	CS6753	4X	
Ga-2	CS6715	3X	L	Kelsterbach-1	CS6100	4X	
Gd-1	CS6716	3X	L	Kelsterbach-1	CS6101	4X	
Ge-1	CS6718	3X	L	Kelsterbach-2	CS6102	3X	L
Gie-0	CS6720	3X	L	Kelsterbach-3	CS6103	4X	
Go-2	CS6722	4X		Kelsterbach-3	CS6104	4X	
Gr-3	CS6725	4X		Kelsterbach-4	CS6105	4X	
Gr-5	CS6727	3X	L	Kil-0	CS6754	3X	L
Gr-6	CS6728	3X	L	Kl-0	CS6756	3X	L

Supplementary Table 1. *CO* genotypes of 271 accessions genotyped for the 7bp indel (continuation).

Accession name	Accession number	promoter type	L311>R substitution	Accession name	Accession number	promoter type	L311>R substitution
Kl-1	CS6757	3X	L	Ma-0	CS6789	4X	
Kl-4	CS6760	3X	L	Ma-2	CS6790	3X	L
Kl-5	CS6761	4X		Me-0	CS1364	4X	
Kn-0	CS6762	3X	L	Mh-1	CS6793	3X	L
Kr-0	CS6764	4X		Mir-0	CS6798	3X	L
Kro-0	CS6766	3X	L	Mrk-0	CS6796	3X	L
La-0	CS6765	4X		Ms-0	CS6797	3X	R
La-1	CS6767	3X	L	Mz-0	CS6800	4X	
Lan-0	CS6768	4X		Na-1	CS6801	3X	L
Lc-0	CS6769	4X		Nc-1	CS6802	4X	
Le-0	CS6770	4X		Nd-0	CS6803	3X	L
Ler-1	CS6928	3X	L	Nie-0	CS6804	3X	L
Li-1	CS6771	3X	L	No-0	CS3081	3X	
Li-10	CS6911	3X	L	Nok-2	CS6809	3X	L
Li-2:1	CS6772	4X		Nok-3	CS6810	3X	L
Li-3	CS1316	4X		Nw-0	CS6811	3X	L
Li-5	CS6775	4X		Nw-1	CS6812	4X	
Li-6	CS6777	3X	R	Nw-2	CS6813	4X	
Li-6:1	CS1328	3X	L	Nw-3	CS6814	4X	
Li-7	CS6778	3X	L	Nw-4	CS6815	3X	L
Li-8	CS6779	4X		Ob-0	CS6816	4X	
Lip-0	CS6780	3X	R	Ob-2	CS6818	4X	
Litva	CS925	4X		Old-1	CS6820	3X	L
Ll-0	CS6781	3X	L	Old-2	CS6821	3X	L
Ll-1	CS6782	3X	L	Or-0	CS6822	4X	
Ll-2	CS6783	3X	L	Ove-0	CS6823	3X	L
Lm-2	CS6784	3X	L	Oy-0	CS6824	3X	L
Lo-2	CS6786	3X	L	Oy-1	CS6929	3X	L
Lu-1	CS1352	3X	L	Pa-1	CS6825	3X	L
Lz-0	CS6788	3X	L	Pa-2	CS6826	3X	L

Supplementary Table 1. *CO* genotypes of 271 accessions genotyped for the 7bp indel (continuation).

Accession name	Accession number	promoter type	L311>R substitution	Accession name	Accession number	promoter type	L311>R substitution
Pa-3	CS6827	4X		Pla-3	CS6836	3X	L
Petergof	CS926	3X	L	Pla-4	CS6837	3X	L
Pf-0	CS6831	4X		Pn-0	CS6838	3X	L
PHW-11	CS6058	3X	L	Po-0	CS6839	3X	L
PHW-13	CS6060	3X	L	Po-1	CS6840	4X	
PHW-15	CS6062	3X	L	Pr-0	CS6841	4X	
PHW-16	CS6065	3X	L	Pt-0	CS6843	3X	L
PHW-2	CS6044	4X		Rak-2	CS6846	4X	
PHW-21	CS6073	3X	L	Rd-0	CS6845	4X	
PHW-22	CS6075	3X	L	RLD1	CS913	4X	
PHW-22	CS6076	3X	L	Rld-2	CS6927	4X	
PHW-24	CS6078	3X	L	Rou-0	CS6847	3X	L
PHW-25	CS6079	3X	L	Rsch-0	CS6848	4X	
PHW-28	CS6083	4X		Rsch-4	CS6850	3X	L
PHW-29	CS6085	3X	L	Rubezhnoe-1	CS927	3X	R
PHW-3	CS6046	4X		Rubezhnoe-2	CS928	3X	R
PHW-30	CS6087	3X	L	S96	CS914	4X	
PHW-32	CS6090	4X		Sap-0	CS6854	4X	
PHW-33	CS6092	3X	L	Sav-0	CS6856	4X	
PHW-33	CS6093	3X	L	Se-0	CS6852	4X	
PHW-34	CS6095	3X	L	Sei-0	CS6853	3X	L
PHW-35	CS6097	3X	L	Sf-1	CS6855	3X	L
PHW-36	CS6098	3X	L	Sf-2	CS6857	4X	
PHW-37	CS6099	3X	L	Sg-1	CS6858	3X	L
PHW-4	CS6047	3X	L	Sg-2	CS6859	3X	L
PHW-5	CS6048	3X	L	Sh-0	CS6860	4X	
Pi-0	CS6832	3X	L	Si-0	CS6861	3X	L
Pi-2	CS6833	4X		Sn(5)-1	CS6181	4X	
Pla-0	CS6834	3X	L	Sp-0	CS6862	3X	L
Pla-1	CS6835	3X	L	St-0	CS6863	3X	L

Supplementary Table 1. ***CO*** **genotypes of 271 accessions genotyped for the 7bp indel (continuation).**

Accession name	Accession number	promoter type	L311>R substitution
Ste-0	CS6864	4X	
Stw-0	CS6865	3X	R
Su-0	CS1540	3X	L
Ta-0	CS6867	4X	
Te-0	CS6918	3X	L
Ts-6	CS6872	3X	L
Ts-7	CS6873	3X	L
Tu-0	CS6875	3X	L
Tu-1	CS6876	3X	L
Uk-1	CS6879	3X	L
Uk-2	CS6881	4X	
Uk-3	CS6880	4X	
Uk-4	CS6882	4X	
Vi-0	CS6883	4X	
Wa-1	CS6885	4X	
Wc-1	CS6886	3X	L
Wc-2	CS6887	4X	
Wei-0	CS6182	3X	L
Wei-1	CS6925	3X	L
Wl-0	CS6920	3X	L
Wil-1	CS6888	3X	L
Wil-2	CS6889	4X	
Ws	CS915	4X	
Ws-0	CS1602	4X	
Wt-1	CS6892	3X	R
Wt-2	CS6893	3X	R
Wt-4	CS6895	3X	L
Wt-5	CS6896	4X	
Wu-0	CS6897	3X	L
Zu-0	CS6902	3X	L
Zu-1	CS6903	3X	L

Supplementary Table 2. ANOVA Summary Tables

**Bolting in unvernalized plants**					
**Source**	**Nparm**	**DF**	**DFDen**	**F Ratio**	**Prob>F**
***CO* type**	1	1	654.5	96.99	<0.0001
***FRI* allele**	1	1	651.7	411.23	<0.0001
**CO*FRI**	1	1	650.2	32.15	<0.0001
**Family[CO,FRI]**	11	11	649.8	45.50	<0.0001
**Random effect**	**Var Ratio**	**Var Component**	**Std error**	**95% Lower**	**95% Upper**
**Block**	-0.02255	4.62	3.50	-2.24	11.49
**Residual**		204.97	11.40	184.33	229.30
**Total**		209.59			

**Number of leaves in unvernalized plants**					
**Source**	**Nparm**	**DF**	**DFDen**	**F Ratio**	**Prob>F**
***CO* type**	1	1	743	194.45	<0.0001
***FRI* allele**	1	1	741.3	443.66	<0.0001
CO*FRI	1	1	739.5	20.81	<0.0001
**Family[CO,FRI]**	11	11	739.6	60.94	<0.0001
**Random effect**	Var Ratio	Var Component	Std error	95% Lower	95% Upper
**Block**	0.0265	1.83	1.19	-0.50	4.16
**Residual**		69.11	3.59	62.57	76.75
**Total**		70.95			

**Bolting in vernalized plants**					
**Source**	**Nparm**	**DF**	**DFDen**	**F Ratio**	**Prob>F**
***CO* type**	1	1	629.4	52.85	<0.0001
***FRI* allele**	1	1	626.5	207.02	<0.0001
CO*FRI	1	1	629.4	26.13	<0.0001
**Family[CO,FRI]**	11	11	627.6	24.99	<0.0001
**Random effect**	**Var Ratio**	**Var Component**	**Std error**	**95% Lower**	**95% Upper**
**Block**	0.0339	1.41	0.88	-0.32	3.15
**Residual**		41.63	2.35	37.37	46.67
**Total**		43.05			

Supplementary Table 2. ANOVA Summary Tables (continuation)

Number of leaves in vernalized plants					
**Source**	**Nparm**	**DF**	**DFDen**	**F Ratio**	**Prob>F**
***CO* type**	1	1	628.1	199.53	<0.0001
***FRI* allele**	1	1	625.7	242.14	<0.0001
CO*FRI	1	1	627.9	42.73	<0.0001
**Family[CO,FRI]**	11	11	626.7	40.12	<0.0001
**Random effect**	**Var Ratio**	**Var Component**	**Std error**	**95% Lower**	**95% Upper**
**Block**	0.04936	1.52	0.79	-0.03	3.08
**Residual**		30.88	1.74	27.72	34.62
**Total**		32.41			

Supplementary Table 3. Mean flowering times.

	**Bolting time.** Letters indicate significant differences (Tukey-test) between groups (p<0.05).			
	**Genotype**	**Least Square means**	**Standard Error**	**Number of observations**
**vernalized**	*CO* 3X	59.66	0.46	298
	CO 4X	63.43	0.44	360
	fri-	57.82	0.47	263
	FRI-Sf2	65.26	0.42	395
	CO 3X, *fri-*	57.27 [c]	0.63	126
	CO 4X, *fri-*	58.38 [c]	0.61	137
	CO 3X, *FRI-Sf2*	62.06 [b]	0.56	172
	CO 4X, *FRI-Sf2*	69.47 [a]	0.50	223
**unvernalized**	*CO* 3X	48.46	0.93	358
	CO 4X	59.65	0.93	414
	fri-	42.55	0.95	306
	FRI-Sf2	65.56	0.91	466
	CO 3X, *fri-*	40.17 [d]	1.25	152
	CO 4X, *fri-*	44.93 [c]	1.25	154
	CO 3X, *FRI-Sf2*	56.75 [b]	1.19	206
	CO 4X, *FRI-Sf2*	74.37 [a]	1.21	260

Supplementary Table 4. Mean flowering times including 2X allele.

	**Bolting time.** Letters within each highlighted box indicate significant differences (Tukey-test) between groups (p < 0.05).			
	**Genotype**	**Least Square means**	**Standard Error**	**Number of observations**
**vernalized**	*CO* 2X	61.94 [b]	0.49	219
	CO 3X	59.66 [c]	0.44	298
	CO 4X	63.42 [a]	0.42	360
	fri-	57.82	0.42	263
	FRI-Sf2	65.52	0.38	395
	CO 2X, *fri-*	57.82 [a]	0.69	90
	CO 3X, *fri-*	57.27 [a]	0.60	126
	CO 4X, *fri-*	58.37 [a]	0.58	137
	CO 2X, *FRI-Sf2*	66.05 [c]	0.59	129
	CO 3X, *FRI-Sf2*	62.04 [d]	0.53	172
	CO 4X, *FRI-Sf2*	68.47 [b]	0.48	223
**unvernalized**	*CO* 2X	55.78 [b]	1.06	254
	CO 3X	48.45 [c]	0.94	358
	CO 4X	59.67 [a]	0.94	414
	fri-	41.60	0.87	306
	FRI-Sf2	67.67	0.82	466
	CO 2X, *fri-*	39.77 [cd]	1.50	102
	CO 3X, *fri-*	40.15 [d]	1.26	152
	CO 4X, *fri-*	44.89 [c]	1.25	154
	CO 2X, *FRI-Sf2*	71.79 [a]	1.32	152
	CO 3X, *FRI-Sf2*	56.76 [b]	1.20	206
	CO 4X, *FRI-Sf2*	74.46 [a]	1.22	260

Supplementary Table 5. Mean primary leaf numbers including 2X allele.

	**Number of leaves.** Letters within each highlighted box indicate significant differences (Tukey-test) between groups (p<0.05).			
	**Genotype**	**Least Square means**	**Standard Error**	**Number of observations**
**vernalized**	*CO* 2X	30.32 [b]	0.41	219
	CO 3X	30.78 [c]	0.43	298
	CO 4X	37.07 [a]	0.41	360
	fri-	30.32	0.41	263
	FRI-Sf2	38.39	0.38	395
	CO 2X, *fri-*	30.02 [c]	0.64	90
	CO 3X, *fri-*	28.78 [c]	0.56	126
	CO 4X, *fri-*	32.15 [b]	0.54	137
	CO 2X, *FRI-Sf2*	40.38 [a]	0.56	129
	CO 3X, *FRI-Sf2*	32.78 [b]	0.50	172
	CO 4X, *FRI-Sf2*	42.00 [a]	0.46	223
**unvernalized**	*CO* 2X	41.20 [b]	0.62	254
	CO 3X	36.01 [c]	0.55	358
	CO 4X	44.58 [a]	0.54	414
	fri-	32.67	0.54	306
	FRI-Sf2	48.52	0.48	466
	CO 2X, *fri-*	30.38 [d]	0.87	102
	CO 3X, *fri-*	30.94 [d]	0.74	152
	CO 4X, *fri-*	36.69 [c]	0.74	154
	CO 2X, *FRI-Sf2*	52.02 [a]	0.74	152
	CO 3X, *FRI-Sf2*	41.08 [b]	0.66	206
	CO 4X, *FRI-Sf2*	52.47 [a]	0.61	260

Supplementary Table 6. Mean primary leaf numbers

	**Number of leaves.** Letters indicate significant differences (Tukey-test) between groups (p<0.05).			
	**Genotype**	**Least Square means**	**Standard Error**	**Number of observations**
**vernalized**	*CO* 3X	30.77	0.42	298
	CO 4X	37.07	0.41	360
	fri-	30.45	0.44	263
	FRI-Sf2	37.38	0.39	395
	CO 3X, *fri-*	28.76 [c]	0.56	126
	CO 4X, *fri-*	32.15 [b]	0.55	137
	CO 3X, *FRI-Sf2*	32.77 [b]	0.50	172
	CO 4X, *FRI-Sf2*	41.99 [a]	0.46	223
**unvernalized**	*CO* 3X	36.00	0.53	358
	CO 4X	44.58	0.52	414
	fri-	33.82	0.56	306
	FRI-Sf2	46.77	0.49	466
	CO 3X, *fri-*	30.93 [d]	0.74	152
	CO 4X, *fri-*	36.71 [c]	0.73	154
	CO 3X, *FRI-Sf2*	41.08 [b]	0.65	206
	CO 4X, *FRI-Sf2*	52.46 [a]	0.59	260

Supplementary Table 7. *CONSTANS* expression profiles of *Arabidopsis* accessions.

**Accession**	**CS number**	***CO* type**	***FRI* type**	***CO* expression (*Actin* normalized)**
Bor-4	CS22591	4x	fri-	5.28
C24	CS22620	3x	FRI+	2.20
Col-0	CS22620	4x	fri-	6.57
Edi-0	CS22657	3x	FRI+	9.87
Knox-18	CS22567	3x	FRI+	1.00
Lp2-2	CS22594	4x	FRI+	6.11
Lp2-6	CS22595	3x	FRI+	2.63
Lz-0	CS22615	3x	fri-	2.89
Mrk-0	CS22635	3x	fri-	1.24
Ms-0	CS22655	3x	FRI+	2.34
Mt-0	CS22642	4x	fri-	8.79
Oy-0	CS22658	3x	fri-	7.51
Ra-0	CS22632	3x	fri-	11.25
Sf-2	CS6857	4x	FRI+	14.73
Tsu-1	CS22647	3x	fri-	1.89
Van-0	CS22627	3x	FRI+	2.97

Supplementary Table S8. Two-way ANOVA Summary Table for *CONSTANS* expression in *Arabidopsis* accessions as response of CO and FRI allele types

Source	Nparm	DF	Sum of Squares	F Ratio	Prob>F
**Model**		3	79.53	1.89	0.1845
***CO* type**	1	1	65.15	4.65	**0.0519**
***FRI* allele**	1	1	3.62	0.25	0.6201
**CO*FRI**	1	1	20.78	1.48	0.2464
**Error**		12	167.97		
**Total**		15	247.50		

Supplementary Table 9. ANCOVA Summary Table for *CONSTANS* expression in *Arabidopsis* accessions as response of *CO* allele type and *FLC* expression

Source	Nparm	DF	Sum of Squares	F Ratio	Prob>F
**Model**		3	110.32	3.21	0.0615
***CO* type**	1	1	32.55	2.84	0.1173
***FLC* expression**	1	1	4.68	0.41	0.5340
CO*FLC	1	1	4.26	0.37	0.5526
**Error**		12	137.18		
**Total**		15	247.50		
